# LncRNA MIR4435-2HG is downregulated in osteoarthritis and regulates chondrocyte cell proliferation and apoptosis

**DOI:** 10.1186/s13018-019-1278-7

**Published:** 2019-08-06

**Authors:** Yu Xiao, Yucheng Bao, Liang Tang, Li Wang

**Affiliations:** 1 Department of Orthopaedic Surgery, Tianjin Hospital, No.406 Jiefang South Road, Hexi, Tianjin City, 300211 People’s Republic of China; 2 Department of Orthopaedic Surgery, Tianjin Haihe Hospital, 890 Jingu Road, Shuanggang Zhen, Jinnan, Tianjin, 300350 People’s Republic of China

**Keywords:** Osteoarthritis, lncRNA MIR4435-2HG, Proliferation, Apoptosis

## Abstract

**Purpose:**

MIR4435-2HG is an oncogenic lncRNA in gastric cancer and lung cancer. Our preliminary microarray data showed that MIR4435-2HG was downregulated in osteoarthritis plasma specimen, indicating the possible involvement of MIR4435-2HG in osteoarthritis.

**Results:**

MIR4435-2HG was downregulated in plasma of osteoarthritis than in plasma of healthy controls. Reduced levels of MIR4435-2HG expression effectively distinguished osteoarthritis patients from the control group. Expression levels of MIR4435-2HG increased after treatment. Overexpression of MIR4435-2HG promoted, while MIR4435-2HG knockdown inhibited the proliferation of chondrocytes. In contrast, MIR4435-2HG overexpression inhibited, while MIR4435-2HG knockdown promoted the apoptosis of chondrocytes.

**Conclusion:**

MIR4435-2HG is downregulated in osteoarthritis and regulates chondrocyte cell proliferation and apoptosis.

## Introduction

Osteoarthritis (OA) is a common clinical degenerative joint disease that is characterized by subchondral bone thickening, degradation of articular cartilage, and the formation of osteophytes [1]. Osteoarthritis now has become a major problem of public health worldwide, and more than half of people aged older than 65 years are suffering from this disease [2]. OA is associated and is closely correlated with homeostatic imbalance, which results from aging [3]. With the increase in people’s life expectancy, the incidence of osteoarthritis is predicted to be further increased in the near future [4]. Although several risk factors have been characterized for osteoarthritis, the pathogenesis of this disease is still unclear and definitive cure is also not available [5].

Although long non-coding RNAs (lncRNAs) have no protein-coding capacity, there are key players in the development of human diseases [6, 7]. We carried out a genome-wide transcriptome analysis of differentially expressed gene in plasma of osteoarthritis patients. Our preliminary data showed that MIR4435-2HG was downregulated in osteoarthritis (data not shown). MIR4435-2HG has been proved to be an oncogenic lncRNA in gastric cancer and lung cancer [8, 9]. We showed that MIR4435-2HG was downregulated in osteoarthritis and regulated chondrocyte cell proliferation and apoptosis.

## Materials and methods

### Research subjects

A total of 78 osteoarthritis (44 cases of knee joint and 34 cases of hip joint; 40 males and 38 females; 60 to 78 years old; mean age 69.0 ± 6.0 years) patients and 58 healthy volunteers (30 males and 28 females; 60 to 77 years; mean age 68.8 ± 5.9 years) were enrolled in Tianjin Hospital from January 2015 to January 2017 to serve as research subjects. All patients were diagnosed by joint fluid analysis, which revealed the existence of inflammation and X-rays, which showed the narrowing of the space between the bones, an indicator of cartilage loss. Patients’ inclusion criteria: (1) new cases of osteoarthritis (stages 3 and 4) (2) patients provided informed consent. Patients’ exclusion criteria: (1) patients complicated with other diseases, such as chronic inflammatory diseases other than osteoarthritis; (2) patients failed to complete treatment; (3) patients who had been treated before admission or currently under medication. The 58 healthy volunteers received systemic physical examinations at the physical health center of Tianjin Hospital, and all parameters were within the normal range. The research has been carried out in accordance with the World Medical Association Declaration of Helsinki, and this study was approved by the Ethics Committee of Tianjin Hospital.

### Plasma specimens, synovial fluid treatment, and cell line

Blood (5 ml) was extracted from both patients and healthy controls 1 day after admission under fasting condition. Synovial fluid was collected from all patients and controls (33 cases of hip and 25 cases of the knee). All patients received treatments including exercise, reducing joint burden, and non-steroidal anti-inflammatory drugs (NSAIDs) such as naproxen. Inflammation was inhibited after 1-month treatment, which was revealed by joint fluid analysis. Blood (5 ml) was also extracted at 1 and 3 months after treatment from each patient under fasting condition. Blood samples were used to prepare plasma samples.

Thirteen cases of synovial membrane from osteoarthritis-affected joints and 8 cases of synovial membrane from non-osteoarthritis-affected joints were obtained from the specimen library of Tianjin Hospital.

Primary chondrocytes (402-05A, Sigma-Aldrich) were used in this study to perform in vitro experiments. Cell culture was performed according to the instructions from Sigma-Aldrich.

### QPCR

To detect the expression of lncRNA MIR4435-2HG, total RNAs were extracted using Trizol reagent (Invitrogen, USA). After reverse transcription (SuperScript IV Reverse Transcriptase kit, Thermo Fisher Scientific., lnc.), SYBR® Green Quantitative RT-qPCR Kit (Sigma-Aldrich) was used to prepare qPCR mixtures. Primers of MIR4435-2HG and endogenous control GAPDH were from Sangon (Shanghai, China). Sequences of primers were: 5′-TGATAAAGGGCTCTGAAAGC-3′ (Forward) and 5′-CACGATGCCTTCACCAGTGT-3′ (reverse) for MIR4435-2HG; 5′-CTGACTTCAACAGCGACAC-3′ (forward) and 5′-TAGCCAAATTCGTTGTCATAC-3′(reverse) for GAPDH. According to 2^−ΔΔCT^ method, expression of MIR4435-2HG was normalized to GAPDH.

### Cell transfection

MIR4435-2HG-expression vector and empty vectors (pEGFP-C3) were from Sangon (Shanghai, China). MIR4435-2HG siRNA and negative control (NC) siRNA were from GenePharma (Shanghai, China). Lipofectamine 2000 reagent (Thermo Fisher Scientific., lnc.) was used for all cell transient transfections with 10 nM vector (empty vector as NC group) or 35 nM siRNAs (NC siRNA as NC group). Cells without transfection were control cells. Cells were collected for subsequent experiments at 24 h after transfection.

### Cell proliferation assay

Cells were collected at 24 h after transfection, and 4 × 10^4^ cells were mixed with 1 ml cell culture medium to make single cell suspensions. Cells were cultivated in a 96-well plate (100 μl each well) at 37 °C, followed by the addition of 10 μl CCK-8 solution (Sigma-Aldrich) at 3 h before the end of cell culture. After that, 10 μl DMSO was added, and the measurement of OD values was performed at 450 nm.

### Cell apoptosis assay

Cells were collected at 24 h after transfection and 4 × 10^4^ cells were mixed with 1 ml serum-free cell culture medium to make single cell suspensions. A 6-well plate was used to cultivate cells (2 ml per well) for 48 h. After that, cells were digested by 0.25% trypsin and stained by Annexin V-FITC (Dojindo, Japan) as well as propidium iodide (PI). Apoptotic cells were separated by flow cytometry.

### Statistical analysis

All experiments were performed in triplicate manner and data were recorded as mean ± standard deviation. Comparisons of expression levels of MIR4435-2HG between two groups were done by unpaired *t* test. Comparisons of expression levels of MIR4435-2HG in patients between different time points were performed by paired *t* test. Comparisons of cell apoptosis and proliferation data among the three groups were done by ANOVA (one-way) and Tukey test. The diagnostic analysis was performed through ROC curve analysis with healthy controls as true negative cases and osteoarthritis patients as true positive cases. DP < 0.05 was statistically significant.

## Results

### Plasma MIR4435-2HG was downregulated in osteoarthritis patients

**Fig. 1:**
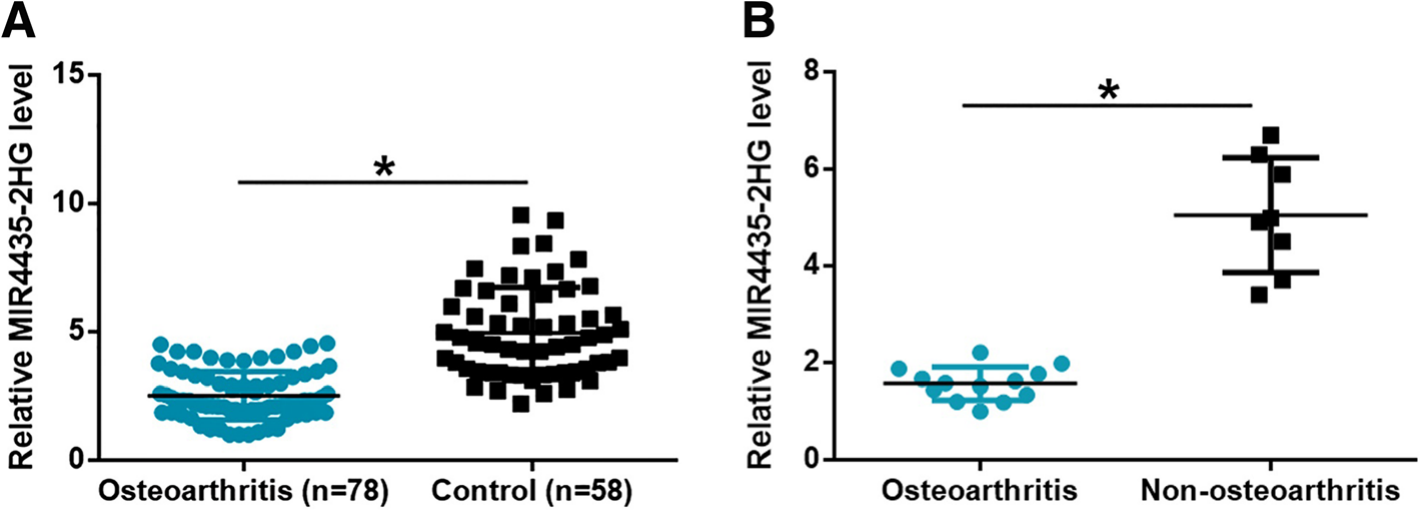
Plasma MIR4435-2HG was downregulated in osteoarthritis patients. RT-qPCR results showed that plasma levels of MIR4435-2HG in osteoarthritis patients and in healthy controls (**a**). In addition, MIR4435-2HG expression in 13 cases of synovial membrane from osteoarthritis-affected joints was also downregulated comparing to 8 cases of synovial membrane from non-osteoarthritis-affected joints (**b**). (*p < 0.05)

RT-qPCR was performed to measure levels of MIR4435-2HG in plasma of osteoarthritis patients and healthy controls. As shown in Fig. 1a, plasma levels of MIR4435-2HG were significantly lower in osteoarthritis patients than in the control group (*p* < 0.05). Moreover, MIR4435-2HG expression in 13 cases of synovial membrane from osteoarthritis-affected joints was also downregulated comparing to 8 cases of synovial membrane from non-osteoarthritis-affected joints (Fig. 1b). These data suggest that downregulation of MIR4435-2HG is likely involved in osteoarthritis.

### Downregulation of MIR4435-2HG in join fluid effectively distinguished osteoarthritis patients from the control group

**Fig. 2:**
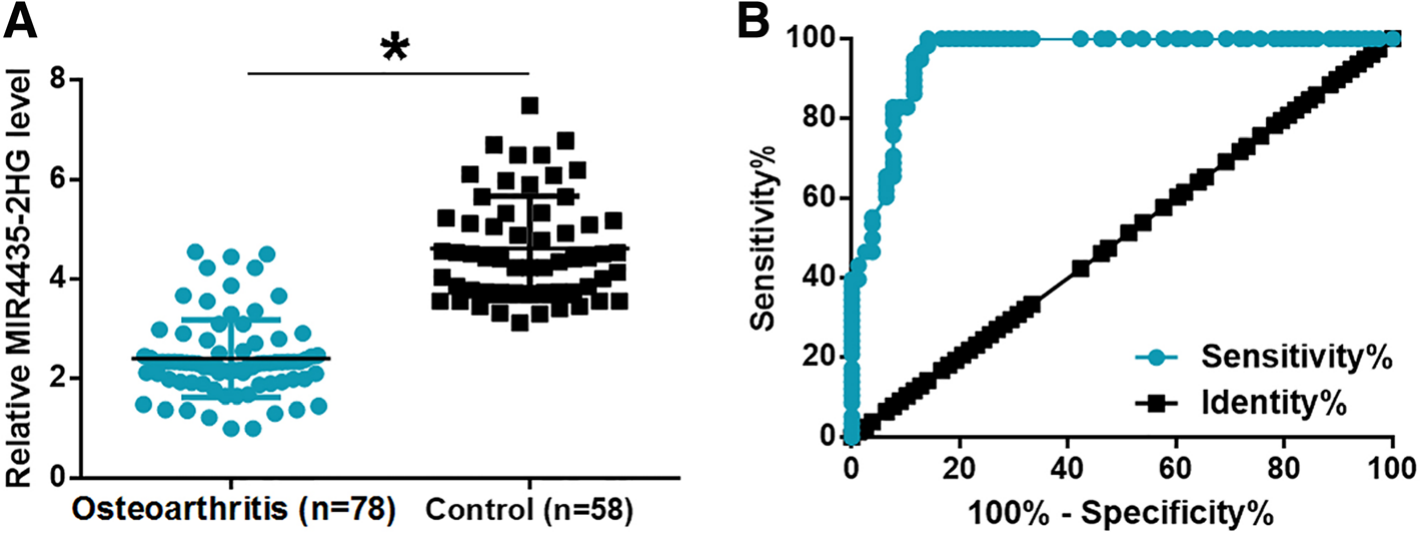
Downregulation of MIR4435-2HG effectively distinguished osteoarthritis patients from healthy controls. MIR4435-2HG in joint fluid was also measured by qPCR. It was observed that expression levels of MIR4435-2HG in join fluid were also significantly lower in osteoarthritis patients than in the control group (**a**). ROC curve analysis showed that downregulation of MIR4435-2HG effectively distinguished osteoarthritis patients from healthy controls (**b**). (*p < 0.05)

MIR4435-2HG in join fluid was also measured by qPCR. It was observed that expression levels of MIR4435-2HG in join fluid were also significantly lower in osteoarthritis patients than in the control group (Fig. 2a, *p* < 0.05). The diagnostic analysis was performed through ROC curve analysis. As shown in Fig. 2, area under the curve was 0.96, with a standard error of 0.016 and 95% confidence interval of 0.92–0.99 (Fig. 2b, *p* < 0001). It is suggested that downregulation of MIR4435-2HG may serve as a potential diagnostic biomarker for osteoarthritis.

### Expression levels of MIR4435-2HG increased after treatment

**Fig. 3:**
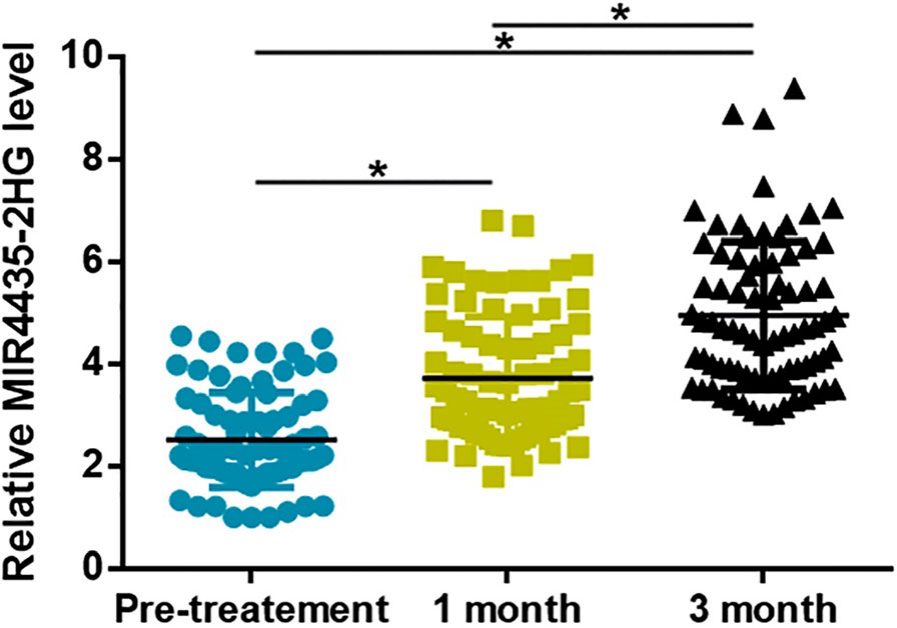
Expression levels of MIR4435-2HG increased after treatment. RT-qPCR results showed that expression levels of MIR4435-2HG increased significantly after treatment (*p < 0.05)

Plasma levels of MIR4435-2HG were measured before treatment as well as 1 and 3 months after the beginning of treatment. Compared with pretreatment levels, plasma levels of MIR4435-2HG were significantly increased at 1 and 3 months after the beginning of treatment (Fig. 3, *p* < 0.05). Comparing to plasma levels of MIR4435-2HG at 1 after the beginning of treatment, plasma levels of MIR4435-2HG were significantly increased at 3 months after the beginning of treatment (Fig. 3, *p* < 0.05).

### MIR4435-2HG regulates the proliferation and apoptosis of chondrocytes

**Fig. 4:**
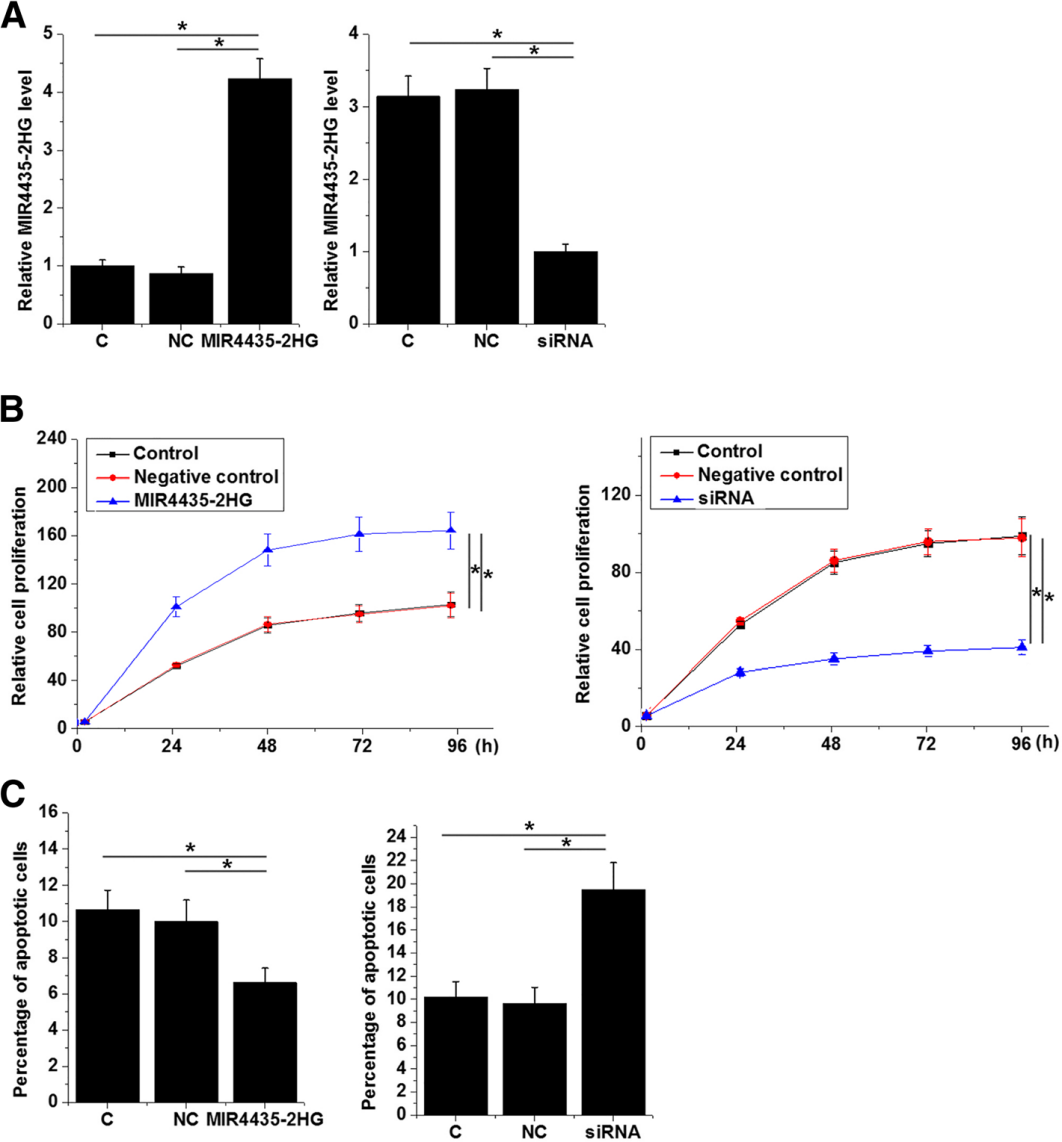
MIR4435-2HG regulates the proliferation and apoptosis of chondrocytes. Overexpression and knockdown of MIR4435-2HG were reached after transfection into chondrocytes (**a**). Overexpression of MIR4435-2HG promoted, while MIR4435-2HG knockdown inhibited the proliferation of chondrocytes (**b**). In contrast, MIR4435-2HG overexpression inhibited, while MIR4435-2HG knockdown promoted the apoptosis of chondrocytes (**c**) (*p < 0.05)

Overexpression and knockdown of MIR4435-2HG were reached after transfection in chondrocytes (Fig. 4a, *p* < 0.05). Overexpression of MIR4435-2HG promoted, while MIR4435-2HG knockdown inhibited the proliferation of cells of chondrocytes (Fig. 4b, *p* < 0.05). In contrast, MIR4435-2HG overexpression inhibited, while MIR4435-2HG knockdown promoted the apoptosis of chondrocytes (Fig. 4c, *p* < 0.05). Therefore, MIR4435-2HG regulated chondrocyte cell proliferation and apoptosis.

## Discussion

Osteoarthritis is a common clinical degenerative joint disease with unknown causes. We found that MIR4435-2HG was downregulated in osteoarthritis and the overexpression of MIR4435-2HG may regulate the behaviors of chondrocytes.

Although the pathogenesis of osteoarthritis is still unclear, the development and progression of this disease is always accompanied by changes in expression pattern of a large set of genes [10, 11], such as lncRNAs Several differentially expressed lncRNAs, such as lncRNA lncRNA HOTAIR and lncRNA UFC1 have been proved to be essential players in osteoarthritis [12, 13]. LncRNA HOTAIR promotes the apoptosis of chondrocytes, which in turn aggregates disease condition [12]. In contrast, lncRNA UFC1 was downregulated in osteoarthritis, and the upregulation of lncRNA UFC1 promotes the proliferation of chondrocyte [13]. Our study first reported the downregulation of plasma circulating MIR4435-2HG could be used to distinguish osteoarthritis patients from the control group. Therefore, plasma MIR4435-2HG may serve as a potential therapeutic biomarker for osteoarthritis. However, more clinical studies are needed to further investigate the diagnostic sensitivity and specificity.

Cartilage cellularity, which is critical for joint function, is reduced in osteoarthritis [14]. It is known that the reduced cartilage cellularity in osteoarthritis patients is closely correlated with chondrocyte death [15, 16]. Chondrocyte apoptosis inhibition is a potential approach for the treatment of osteoarthritis [17]. In the present study, we showed that MIR4435-2HG promoted the proliferation but inhibited the apoptosis of chondrocytes. Therefore, overexpression of MIR4435-2HG may serve as a promising therapeutic target for the treatment of osteoarthritis. However, more clinical trials and in vivo animal model studies are needed to further test our hypothesis.

## Conclusion

In conclusion, MIR4435-2HG is downregulated in osteoarthritis and may serve as a therapeutic target for osteoarthritis by promoting chondrocyte cell proliferation and inhibiting cell apoptosis.

## Data Availability

The analyzed datasets generated during the study are available from the corresponding author on reasonable request.

## Abbreviations

lncRNAs: Long non-coding RNAs
NSAIDs: Non-steroidal anti-inflammatory drugs
OA: Osteoarthritis
